# *In situ* electrochemical activation as a generic strategy for promoting the electrocatalytic hydrogen evolution reaction and alcohol electro-oxidation in alkaline medium†

**DOI:** 10.1039/d0ra07817d

**Published:** 2021-03-12

**Authors:** Alireza Kardan, Narges Ashraf, Zeynab Dabirifar, Sara Khadempir

**Affiliations:** Department of Chemical Engineering, Quchan University of Technology Quchan Iran s.khadempir@qiet.ac.ir; Department of Chemistry, Faculty of Science, Ferdowsi University of Mashhad Mashhad Iran

## Abstract

*In situ* electrochemical activation as a new pre-treatment method is extremely effective for enhanced electrocatalytic performances for different applications. With the help of this method, *in situ* surface modification of electrocatalyst is achieved without using pre-made seeds or complex synthesis procedure. Herein, with the purpose of finding an *in situ* and simple electrochemical activation protocol, the green synthesis of Au/Pd nanoparticles (AuPd) by means of polyoxometalate (POM) is reported. Structural analysis of the AuPd nanohybrid unveil the Au-core/Pd-shell structure which surrounded by POM. We propose a novel cathodic electrochemical activation in phosphate buffer solution which can greatly boost the electrocatalytic activity of the as-prepared AuPd and Pd electrocatalyst not only for hydrogen evolution reaction (HER) as a model of electro-reduction, but also for methanol and ethanol electro-oxidation reaction (MOR & EOR). For the HER in 1 M NaOH solution, after the electrochemical activation, the needed potential to drive a geometrical current density of 10 mA cm^−2^ significantly decreases from – 400 mV *vs.* the reversible hydrogen electrode (RHE) to −290 mV *vs.* RHE. For the EOR and MOR, electrochemically activated AuPd realized 3.4- and 2.9- fold increase in mass current density (mA mg_Pd_^−1^) with respect to the pristine AuPd electrocatalyst, respectively.

## Abstract 2

*In situ* electrochemical activation as a new pre-treatment method is extremely effective for enhanced electrocatalytic performances for different applications.

## Graphical abstract

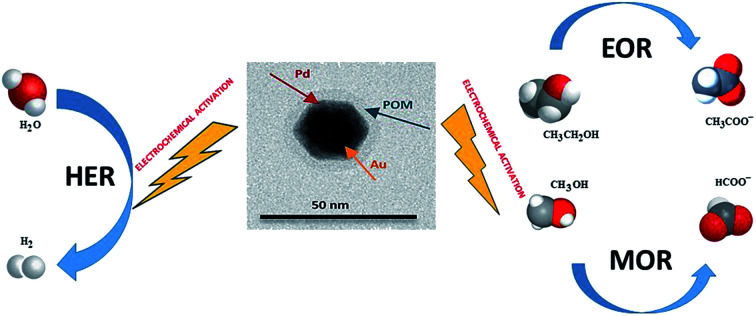

## Introduction

The world energy consumption growth and global environmental crisis create serious demand for replacing fossil fuels – as the main non-renewable source of energy around the world – with renewable energy sources like solar and wind.

However, the large scale construction of renewable energy systems is still challenging due to technical barriers.^1–4^ Challenges such as reliability, durability and energy storage have a significant effect on the development of renewable energy technologies. At present, the key obstacle is “energy storage”, and one way to address the solution is converting electrical energy into a stable chemical product:^3,5,6^ for example, electrochemical water-splitting systems produce hydrogen gas and breathable oxygen *via* (eqn (1)) with the aid of electricity that comes from renewable sources energy.

1 2H_2_O (l) → 2H_2_ (g) + O_2_ (g)

H_2_ is an attractive clean fuel and energy carrier owing to its high mass energy density. In these systems, the electrocatalyst-coated electrodes are inserted into water and powered by electricity. The interaction of the electricity, the electrocatalyst and water generate H_2_*via* hydrogen evolution reaction (HER), and O_2_ through oxygen evolution reaction (OER).^7,8^

Moreover, direct alcohol fuel cells (DAFCs) consider to be a relevant technology to generate electricity *via* the electro-oxidation of low molecular weight alcohols such as methanol and ethanol. Meanwhile, to enable alcohol based fuel cells, one of the main issues is the requirement of useful electrocatalysts.^9–13^

Over the past decades, particular attention has been paid to design and development of efficient and low-cost electrocatalysts that are used in water-splitting systems and DAFCs. Different approaches has been suggested to improve the performance of these electrocatalysts, which include the use of a conductive support, incorporation of a second element, or employment of pre-treating methods such as electrochemical activation.^14–16^

*In situ* electrochemical activation (EA) has remarkable potential to obtain much greater electrocatalytic performances. The promising EA techniques generally involve simple electrochemical methods such as cathodic reduction,^17,18^ anodic oxidation,^19,20^ electrochemical incorporation,^21,22^ and electro-dissolution.^23,24^ According to this concept, surface of the electrocatalyst is modified by generation of active species which can promote the electron transfer kinetics. Various research groups have explored diverse EA methods and their associated mechanisms to develop electrocatalytic activity.^25,26^ However, further insights are still required to provide more information about EA processes.

In this study, we introduce an innovative *in situ* cathodic EA approach to enhance the electrocatalytic activity of AuPd core–shell nanoparticles as a vastly used electrocatalyst for diverse important electrocatalytic reaction such as hydrogen evolution and oxidation reactions (HER/HOR), oxygen evolution and reduction reactions (OER/ORR), alcohol electro-oxidation reactions, and so on. Based on the green chemistry rules, introducing environmentally friendly multifunctional substrates is a promising aspect.^27,28^ AuPd were synthesized using an environmentally friendly one-pot approach with the help of polyoxometalate (POM) as dual cooperative reducing and stabilizing agent. The proposed *in situ* cathodic EA process is applied to improve the electrocatalytic activity of AuPd not only towards HER as an electro-reduction representative, but also towards ethanol and methanol electro-oxidation reactions (EOR & MOR). Through the detailed electrochemical analysis, we disclose that the EA process has a significantly boosted effect on HER, EOR and MOR *via* the surface modification of the as-prepared electrocatalysts and would render as a generic strategy for fabrication of the advanced electrocatalysts for future practical applications.

## Experimental section

### Chemicals

Tetrachloroauric(III) acid trihydrate (HAuCl_4_·3H_2_O), palladium(ii) chloride (PdCl_2_), sodium hydroxide (NaOH), isopropyl alcohol (C_2_H_6_OH), ethanol (C_2_H_5_OH), methanol (CH_3_OH), sodium dihydrogen phosphate dihydrate (NaH_2_PO_4_·2H_2_O), sulphuric acid (H_2_SO_4_) were obtained from Merck (Darmstadt, Germany). Phosphomolybdic acid (H_3_PMo_12_O_40_ – denoted as PMo_12_) was supplied from Sigma-Aldrich. Phosphate buffer solution (PBS, 0.1 M) was prepared by dissolving the appropriate amount of NaH_2_PO_4_·2H_2_O in deionized (DI) water and adjusting the pH to 8.4 by 0.1 M NaOH aqueous solution.

### Synthesis of nanocomposites

For the synthesis of Au@Pd/PMo_12_ (denoted as AuPd), 0.67 mg PdCl_2_ and 27.4 mg PMo_12_ were put into 100 mL DI water, followed by addition of 1.6 mg HAuCl_4_·3H_2_O (atomic ratio of Au to Pd was adjusted to 1 : 1). Then, 10 mL of the as-prepared solution was put into a spectrophotometer cell. Subsequently, 2 mL isopropyl alcohol was added and the reaction mixture was irradiated by a UV mercury-vapor lamp (125 W) and continuously reacting for 2 h. The AuPd material is separated from the solution by centrifuging and washing with DI water for several times to remove any possible remaining ions. The collected nanocomposite is then dried for different form of characterization and catalytic activity studies. The Pd/PMo_12_ (denoted as Pd) nanocomposite was produced with the same process without addition of Au salt solution and also Au/PMo_12_ (denoted as Au) was synthesized by the same process without addition of Pd salt solution.

### Characterization

Nanocomposites were characterized with the help of scanning electron microscopy (SEM), transmission electron microscopy (TEM), high-angle annular dark-field scanning TEM (HAADF-STEM) and X-ray diffraction (XRD). The composition of the nanocomposite was determined by inductively coupled plasma mass spectrometry (ICP-MS). The –OH increments of AuPd were checked by Fourier transform infrared spectrometry (FT-IR).

### Working electrode fabrication

The catalyst ink was made as under below: the suitable amount of the as-prepared catalyst powder was dispersed in DI water and treated with ultrasound for 20 minute to obtain the 1.0 mg mL^−1^ aqueous suspension. The working electrode would be fabricated by drop casting the 3 μL of catalyst ink onto previously polished glassy carbon electrode (GCE, 2 mm in diameter, Azar electrode Co.) with 0.05 μm alumina slurry. Then, the modified GCE was dried under IR lamp.

### Electrochemical measurements

A μ-Autolab type III Potentiostat was employed to conduct the electrochemical experiments. A three-compartment electrochemical cell was fabricated using Ag/AgCl as the reference electrode, platinum wire as the counter electrode and modified GCE as the working electrode. Three electrochemical techniques were applied to investigate the electrochemical behaviour of electrodes containing linear sweep voltammetry (LSV), cyclic voltammetry and chronoamperometry.

LSV was performed to test the HER activity in alkaline medium (1 M NaOH aqueous solution) at a scan rate of 50 mV s^−1^.

EOR and MOR were conducted by cyclic voltammetry in alkaline medium from −0.8 V to 0.4 V (*vs.* Ag/AgCl) at 50 mV s^−1^.

The chronoamperometry test with a fixed potential of −1.3 V (*vs.* Ag/AgCl) and 300 second duration in PBS (0.1 M, pH 8.4) was used to *in situ* electrochemical activation of the as-prepared electrocatalyst. Then the electro activated catalyst (hereafter called EA-catalyst) was washed with water and will be ready for further electrochemical investigations.

All potentials in HER study were referenced to a reversible hydrogen electrode (RHE) by the following equation:^29^*E*_RHE_ = *E*_Ag/AgCl_ + 0.059 × pH + *E*^0^_Ag/AgCl_

## Results and discussion

### Characterization experiments

**Fig. 1:**
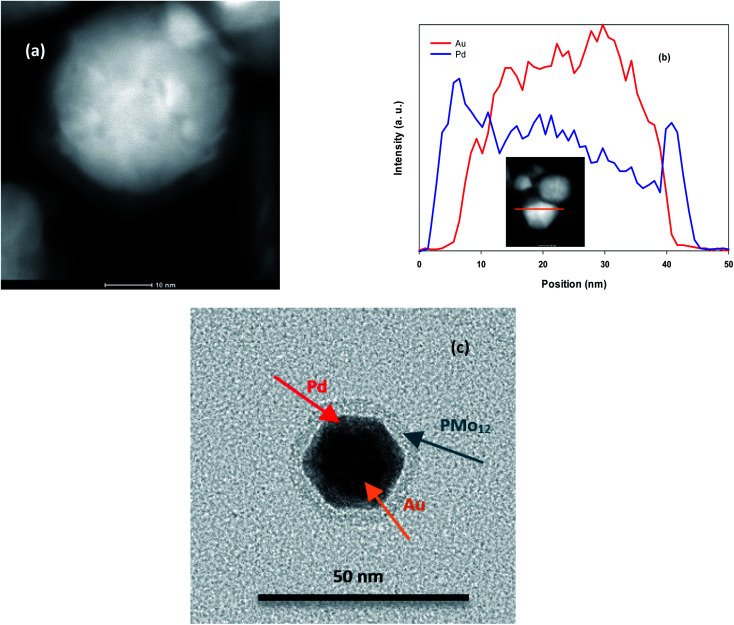
(a) HAADF-STEM image of AuPd, (b) EDS line scan profile. The inset in (b) corresponds to the HAADF-STEM image of AuPd, (c) TEM image of AuPd.

HAADF-STEM image of AuPd (Fig. 1a) shows a clear contrast between the brighter and darker layer that verifies the core@shell structure. Also, SEM (Fig. S1†) and TEM images (Fig. S2†) of AuPd represent nanoparticles with diameter of about 25 nm. Moreover, cross-sectional EDS line scan profiles (Fig. 1b) obtained from the HAADF-STEM image validate that Au core is surrounded by Pd shell. It is noted that this profile was tuned to determine Pd and Au elements. In addition, individual TEM image of AuPd (Fig. 1c) includes more information. Three separate regions is obviously seen in the image: a dense core at the centre as a dark area designated with Au, then an intermediate shell covering the core labelled Pd, and an outer layer marked PMo_12_ playing the efficient capping role for AuPd. These data are also supported by EDS spectra (Fig. S3†).

**Fig. 2:**
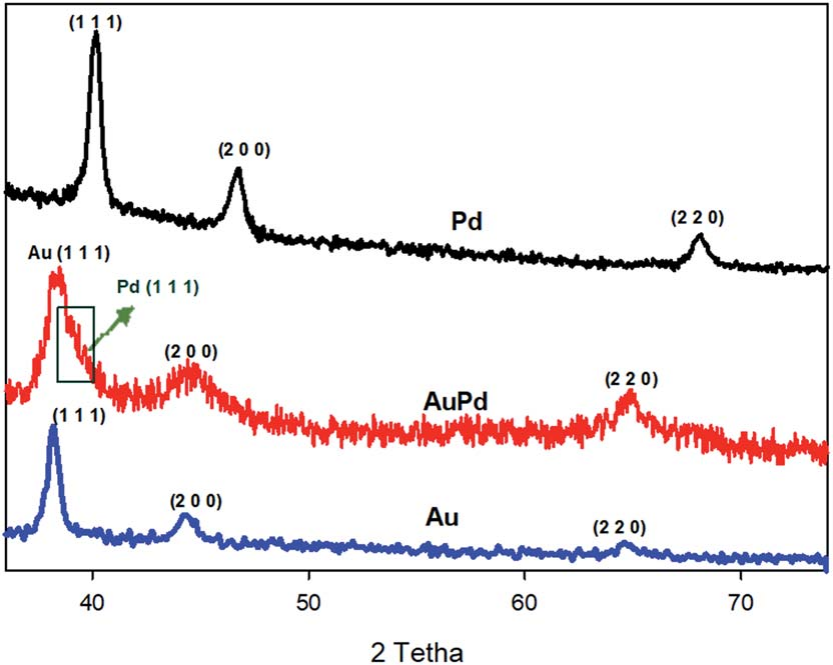
XRD pattern of Pd, Au and AuPd.

The crystalline structure of materials can be checked by XRD analysis. Fig. 2 provides the XRD patterns of the as-made AuPd, Au, and Pd. For both of the Au and Pd, three diffraction peaks correspond to the (1 1 1), (2 0 0), (2 2 0) planes are observed, which can be ascribed to the fcc structure of Au and Pd metals (JCPDS: 01-071-4073 for Au and 01-071-3757 for Pd).^30,31^ Also, the diffraction peaks for the AuPd are attributed to the fcc structure of the metals. When compared to the monometallic nanoparticles, the peak position of bimetallic AuPd are very close to those of the Au rather than the Pd, indicating that the Au core is not significantly strained. Nevertheless, there is an apparent shoulder (marked by green arrow in Fig. 2) that superimposed with the Au (1 1 1) peak, suggesting the very thin layer of the Pd shell. The position of this shoulder – Pd (1 1 1) peak – has been negatively shifted with respect to the XRD pattern of monometallic Pd, which could be related to a lattice expansion of about 2.2% to 2.5%, owing to the large strain induced by lattice mismatch between the Au core and the Pd shell.^32,33^

On the other hand, the characteristic peaks of the PMo_12_ are not detected in the XRD spectra of AuPd, inferring that the PMo_12_ clusters are in the dispersed amorphous state, till in the crystalline state.^34^

Surface properties of nanoparticles can be further characterized by the application of cyclic voltammetry (CV) technique. Fig. S4† depicts the multi-scan cyclic voltammogram of the AuPd modified GCE acquired in 0.5 M H_2_SO_4_ solution. In the cathodic scan of the first cycle, only the Pd oxide reduction peak is visible, *i.e.*, the surface is exposed to Pd atoms. As the cycle number increases, the Au oxide reduction peak also appears and its intensity gradually increases; whilst the intensity of the Pd oxide reduction peak decreases, so the continuous changes in the surface property of the AuPd is happened due to electrochemical dissolution of Pd, which is well documented in acidic media.^35,36^ In other words, continual dissolution of Pd leads to the detection of Au, indicating a core@shell structure where a core of Au atoms is covered by a shell of Pd atoms.

### *In situ* electrochemical activation procedure

To demonstrate the effect of electrochemical activation on the performance of the as-prepared electrocatalysts, HER was considered as a reductive electrochemical process, while MOR and EOR were studied as oxidative processes.

### HER study

**Fig. 3:**
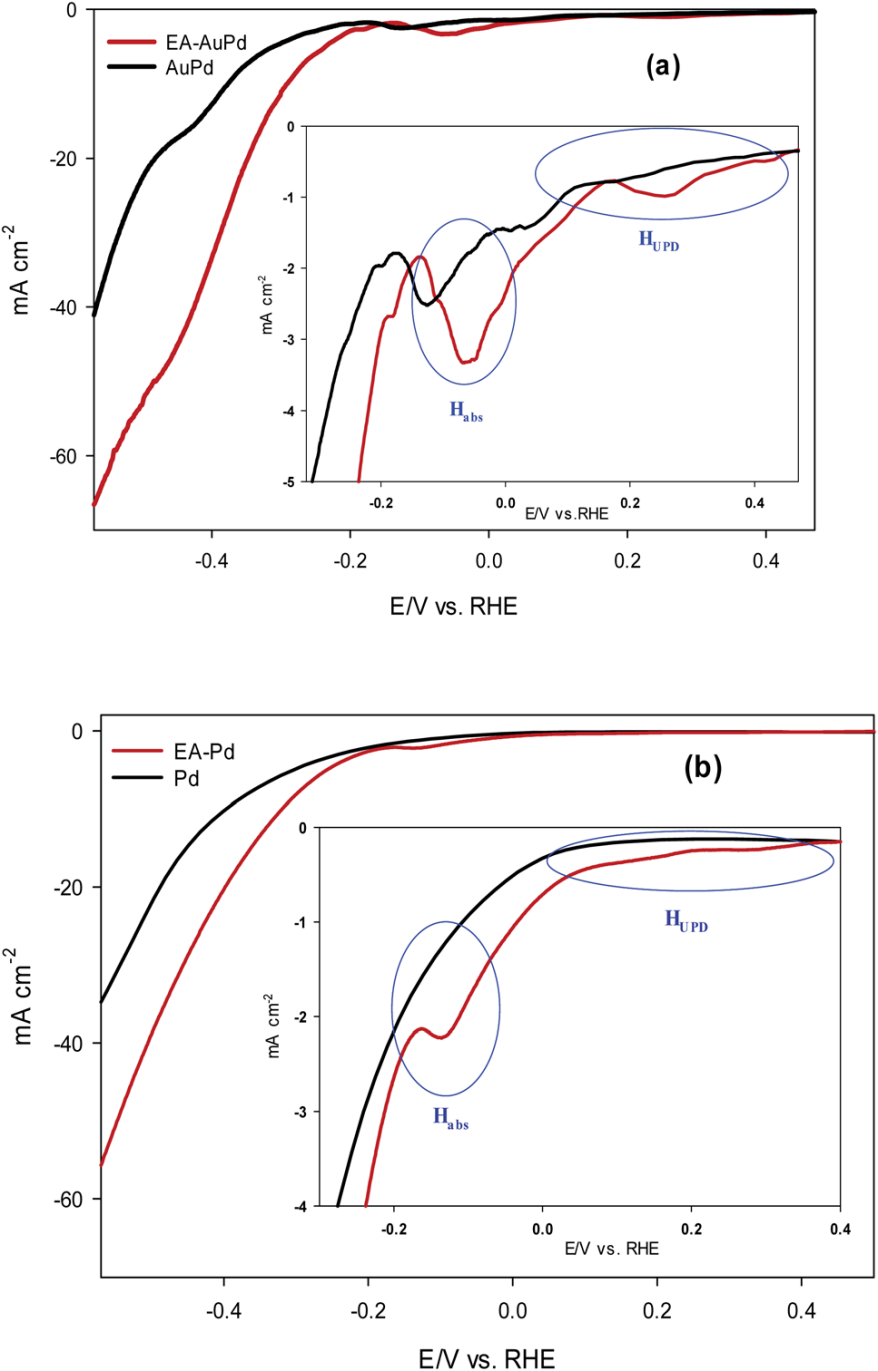
LSV polarization curves for HER on: (a) AuPd and EA-AuPd electrocatalysts, (b) Pd and EA-Pd electrocatalysts. The insets show zoom-in at lower current density. Measurements were conducted in 1 M NaOH solutions at a scan rate of 50 mV s^−1^.

The HER electrocatalytic activity of AuPd and EA-AuPd electrocatalysts were investigated in 1 M NaOH solution using the LSV method and geometric polarization curves (*I*–*V* plot) has been shown in Fig. 3a. In order to evaluate the electrocatalytic performance of the electrocatalysts, the overpotential values corresponding to the current density of 10 mA cm^−2^ (*η*_10_) and 50 mA cm^−2^ (*η*_50_) are considered as important criteria.^37^ For AuPd, *η*_10_ and *η*_50_ are obtained to be −400 and −600 mV; while for EA-AuPd, *η*_10_ and *η*_50_ are −290 and −450 mV, respectively. The same trend was obtained for Pd and EA-Pd as shown in Fig. 3b and also for Pd/C and EA-Pd/C (Fig. S5†). These results verify that the activated electrocatalysts deliver higher cathodic currents compared to the non-activated electrodes at same potentials. Moreover, Tafel slope, another important measure for evaluating the performance of the electrocatalysts, was obtained for AuPd, EA-AuPd, Pd and EA-Pd (Table 1). From the results, it is realized that the Tafel slopes for the activated electrocatalysts are lower than those of the non-activated electrocatalysts, which means that the energy needed to raise the current is lower for activated electrocatalysts. All of the above evidences implies that the HER has boosted by applying the EA process.

**Table 1:** Tafel slopes of electrocatalysts

Tafel slope	Pd	AuPd
Non activated (mV dec^−1^)	280	250
Electro-activated (mV dec^−1^)	245	224

**Fig. 4:**
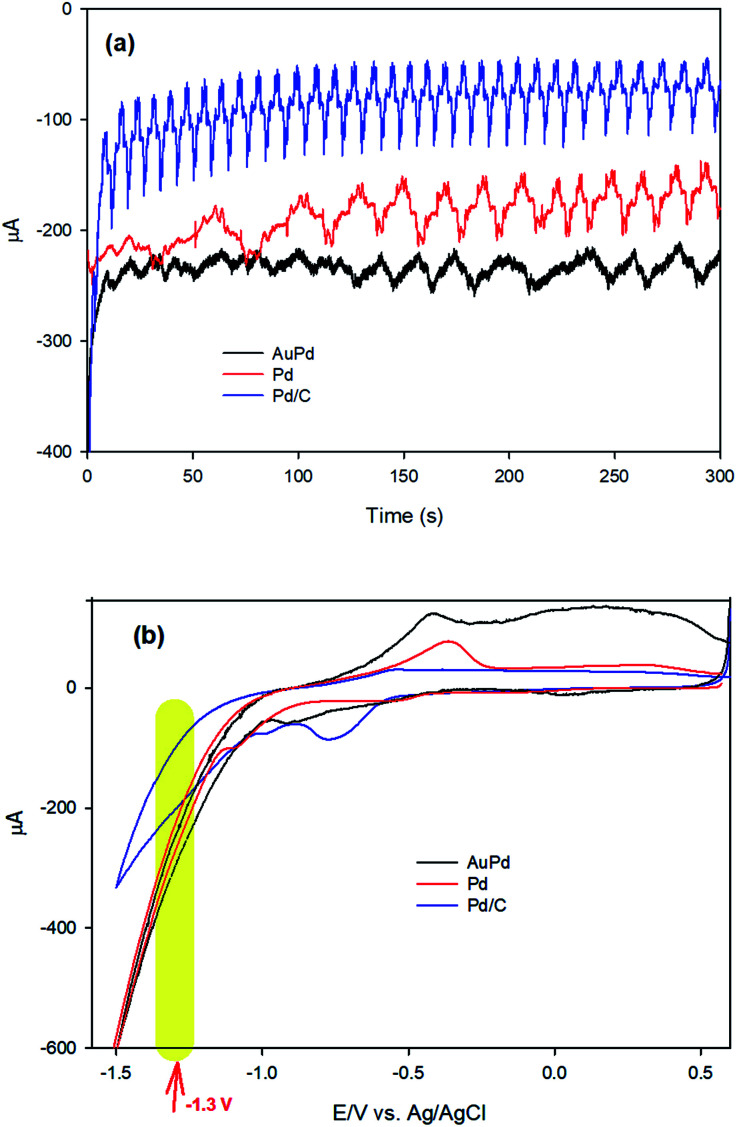
(a) Chronoamperograms of AuPd, Pd and Pd/C at −1.3 V in PBS (pH 8.4), (b) cyclic voltammograms (CV) of AuPd, Pd and Pd/C in PBS (pH 8.4), scan rate: 50 mV s^−1^.

To investigate the mechanism of the EA process, we commence our discussion by analysing the results of chronoamperometry (CA) responses. Fig. 4a shows the chronoamperograms recorded during the EA stage of AuPd, Pd and Pd/C in PBS (pH 8.4), indicating an electrochemical reduction process is carried out at the electrode surfaces. Moreover, CV was employed to further realize what happens throughout the EA stage at the corresponding applied potential (−1.3 V). Fig. 4b represents the CVs of Pd and AuPd within the potential range of 0.6 to −1.5 V in PBS (pH 8.4). The acute increase of cathodic current behind the −1.1 V corresponds to the electrochemical HER in alkaline media, which is supposed to be as eqn (2).^2,37^

2 2H_2_O + 2e^−^ → H_2_ + 2OH^−^

So, it can be concluded that HER occurs at the applied activation potential during the EA process. Therefore, it is valuable to describe what happens during HER in alkaline media.

**Scheme 1:**
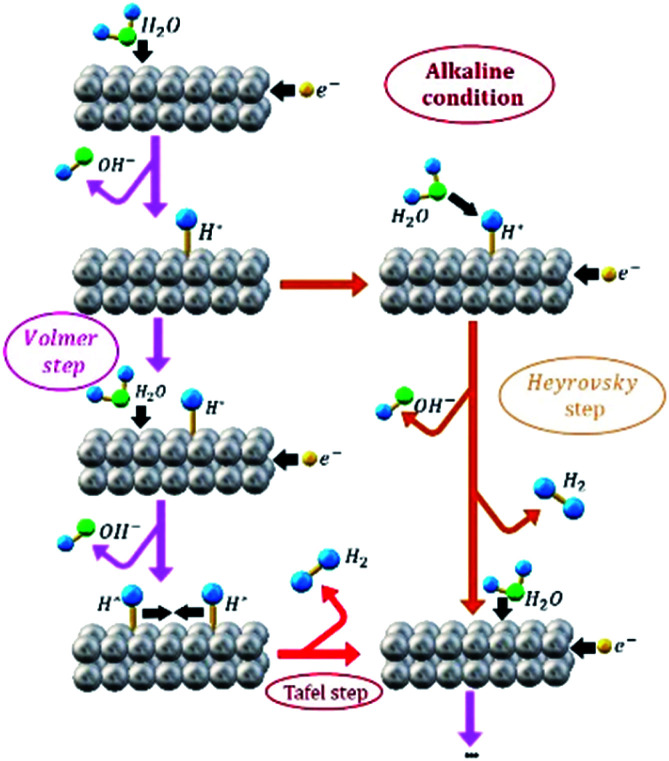
Representation of HER pathway under alkaline condition.

In order to distinguish between the HER that occur during EA process and the HER as an electro-reduction reaction model, from now on these processes will be identified by “EA-HER” and “HER”, respectively. It has been generally accepted that HER consists of three possible reactions. Scheme 1 illustrates the HER pathway under alkaline condition. In the first step, which is called Volmer (eqn (3)), electrochemical dissociation of water molecules – as a unique proton donor in neutral and alkaline media – to the adsorbed H* and OH^−^ intermediates occurs:

3 H_2_O + e^−^ → H*+ OH^−^ (Volmer)

The second step is either Heyrovsky (eqn (4)) or Tafel (eqn (5)) step.

4 H_2_O + H*+ e^−^ → H_2_ + OH^−^ (Heyrovsky step)

5 2H* → H_2_ (Tafel recombination step)

Based on the obtained Tafel slope values that are all more than 120 mV dec^−1^ (Table 1), it can be concluded that Volmer reaction is the rate determining step (r.d.s) in the HER mechanism, including the adsorption of water molecules and cleavage of H–OH bonds at the surface of the AuPd and Pd electrocatalysts in alkaline media, which requires high energy consumption.

**Fig. 5:**
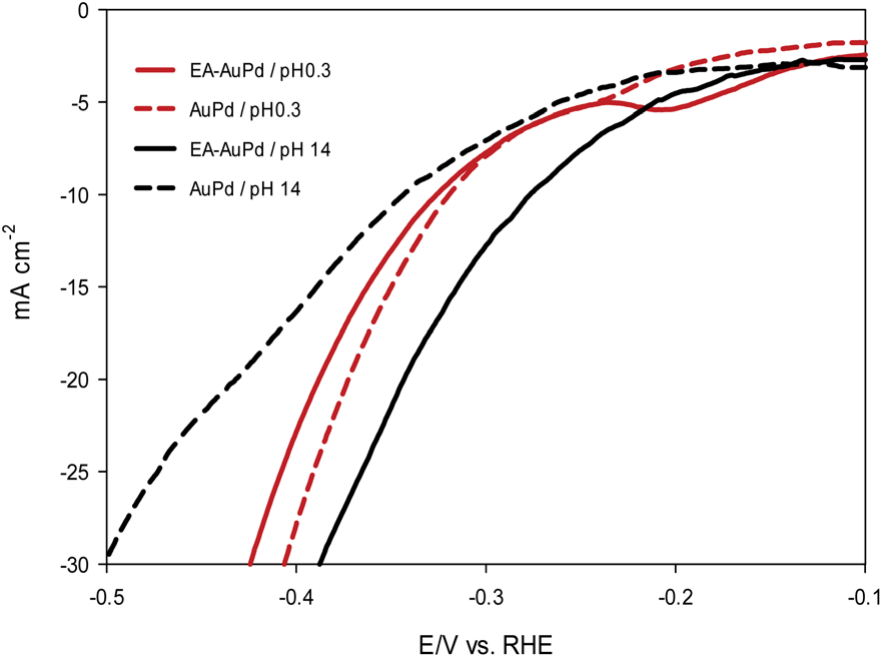
LSV polarization curves of AuPd and EA-AuPd electrocatalyst for the HER. Measurements were conducted in 0.5 M H_2_SO_4_ (pH 0.3) and in 1 M NaOH (pH 14) at a scan rate of 50 mV s^−1^.

Now, it is useful to consider the focused LSVs before and after the EA process (see inset of Fig. 3a and b). The LSVs consist of three distinct areas: (i) the adsorption of underpotentially deposited hydrogen (H_UPD_) which is the hydrogen atoms adsorbed at more positive potentials predicted by Nernst equation for the hydrogen reaction, (ii) the absorption of hydrogen atoms into the palladium lattice (H_abs_), and (iii) H_2_ evolution at last.^39–43^ The inset in Fig. 3a and b demonstrate that all of the above three processes are facilitated after EA step. The evidence for this can be sought in the fact that the features of the LSVs differ clearly before and after the EA process. A quick glance at the LSVs reveals that the signals corresponding to H_UPD_, H_abs_ and H_2_ evolution have shifted towards more positive potentials and become sharper compared to the poorly defined signals before the EA process. It should be noted that the features of H_UPD_ signal is under the influence of the Volmer reaction. The more Volmer reaction is facilitated, the more H_UPD_ signals shifts towards positive potentials. Therefore, by comparing the signals attributing to H_UPD_ before and after the EA process, it can be concluded that the kinetics of Volmer reaction has considerably improved after the EA process. The reason for this facilitation can be explained by considering that during EA-HER, OH^−^ ions are generated and captured within the nanoscopic cavities of the electrocatalyst and entrapped such that they cannot be removed even by washing.^44^ These entrapped OH^−^ ions (denoted as Pd/OH^−^_ent_) provide active sites for adsorption and cleavage of H–OH bonds by establishment of the Pd/OH^−^_ent_⋯OH_2_ activated complex. Indeed, hydrogen bonding between Pd/OH^−^_ent_ and H_2_O promotes the surface hydrophilicity and lowers the energy barrier for water dissociation that results in higher electrocatalytic activity of EA-AuPd for the HER.^38,45–47^ To further evaluate this hypothesis, the HER electrocatalytic activities of the EA-AuPd and AuPd were also investigated in acidic media (Fig. 5). On the basis of experimental results, it is revealed that in acidic medium in which the proton donor is H_3_O^+^ instead of H_2_O, the electrocatalytic activity of EA-AuPd and AuPd do not differ substantially; since the formation of Pd/OH^−^_ent_⋯OH_2_ complex is irrelevant.^38^

### MOR and EOR study

**Fig. 6:**
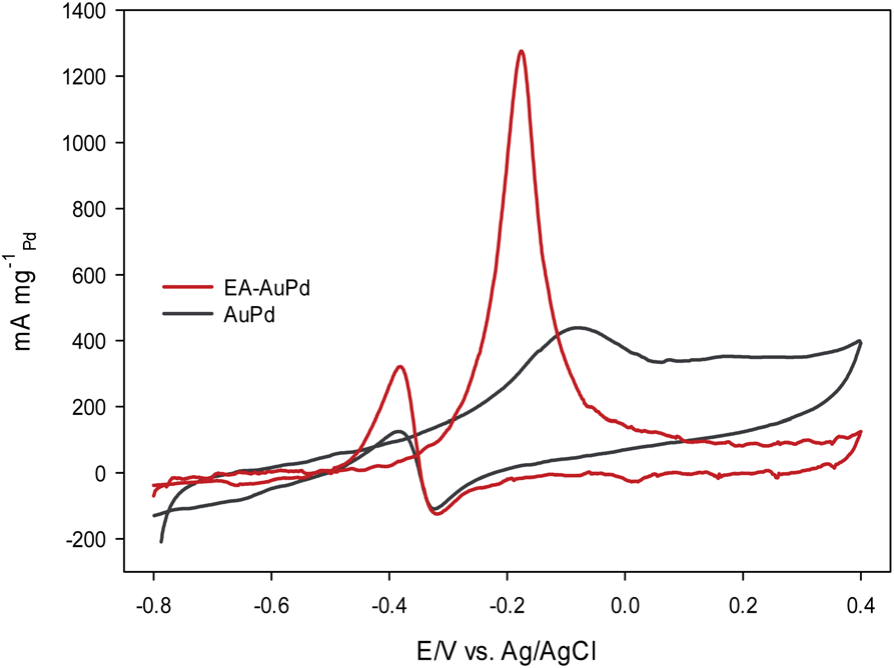
CV curves of AuPd and EA-AuPd in 1 M NaOH + 1 M methanol at a scan rate of 50 mV s^−1^.

**Fig. 7:**
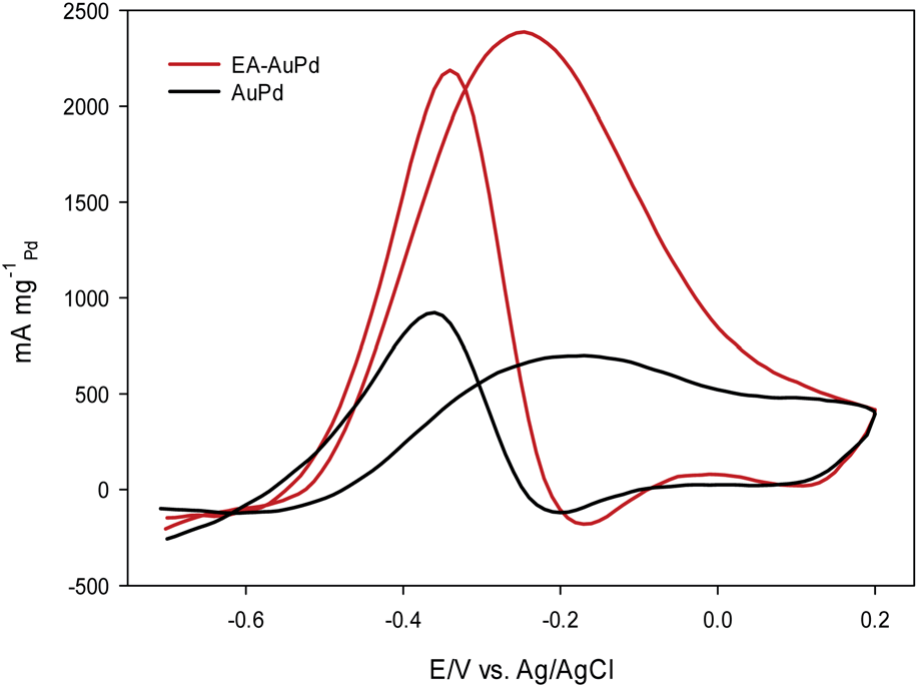
CV curves of AuPd and EA-AuPd in 1 M NaOH + 1 M ethanol at a scan rate of 50 mV s^−1^.

Fig. 6 and Fig. 7 show the CVs of EA-AuPd and AuPd in methanol and ethanol containing alkaline solutions, respectively. The features of CVs for the MOR and EOR under alkaline conditions are matched to the literature reports.^48–51^ The anodic current peak in the forward scan is assigned to the oxidation of freshly chemisorbed small alcohol (methanol or ethanol) molecule. There is an ambiguity about the origin of cathodic current peak, such that some authors attributed it to the oxidation of alcohol,^52,53^ while the others ascribed this to the removal of carbonaceous chemicals intermediates that are generated during the anodic scan.^51,54^ Therefore, we focus on the anodic peak as a basis for comparison of catalytic activity in the following sections.

It was perceived that the anodic peak current densities in the forward sweep are 1270 mA mg^−1^_Pd_ and 440 mA mg^−1^_Pd_ for EA-AuPd for AuPd, respectively, showing that the catalytic activity of EA-AuPd is 2.9 times greater than that of AuPd for MOR. Also, the MOR anodic peak potential for EA-AuPd is about 100 mV lower than that for AuPd. Through the use of EA-process the same results were also obtained for EOR. Fig. 7 indicates that the electrocatalytic activity of EA-AuPd is 3.4 times greater than that of AuPd. The similar outcomes were acquired for Pd, and Pd/C (Fig. S6 and S7†).

**Fig. 8:**
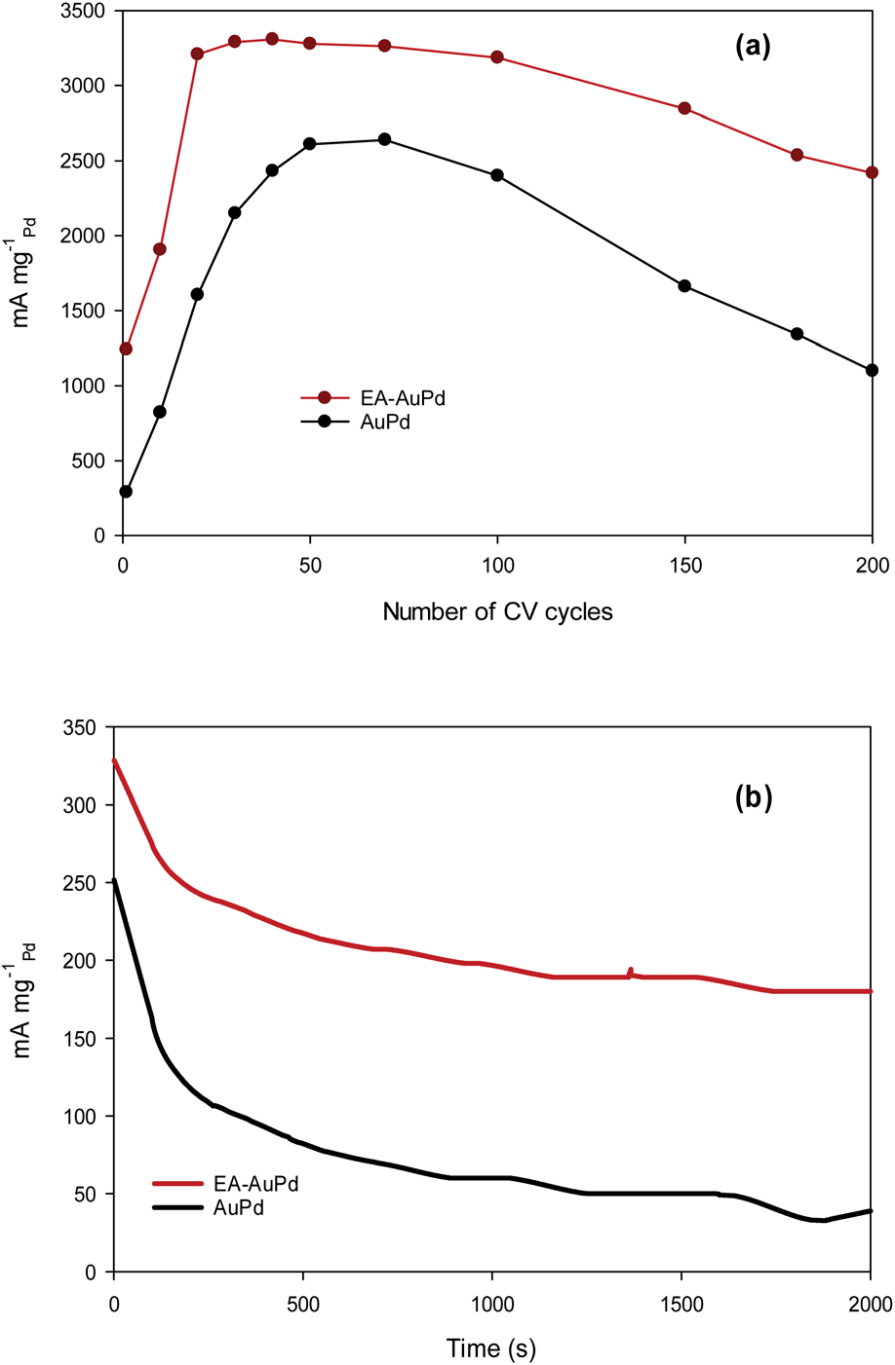
(a) Dependence of anodic current peak for EA-AuPd and AuPd catalysts on cycle number. (b) Chronoamperometry curves of EA-AuPd and AuPd catalysts for the MOR measured at −0.18 V (*vs.* Ag/AgCl). All experiments were conducted in 1 M methanol + 1 M NaOH.

Besides the electrocatalytic activity, the durability (long-term stability) is still a crucial challenge in material development for long-term practical applications. To examine this purpose, accelerated durability test (ADT) and chronoamperometric measurement were conducted at the EA-AuPd and AuPd electrocatalysts.^55^ The ADT for the electrodes was performed by applying successive CV aging (200 cycles) from −0.8 V to 0.4 V (*vs.* Ag/AgCl) in the presence of methanol and NaOH solution (Fig. S8a and b†). For both EA-AuPd and AuPd, the initial increase in the anodic peak currents are observed which is assigned to the more accessible active sites. Also, Fig. 8a shows the dependence of the anodic peak current on the cycle number (obtained from Fig. S8a and b†). For EA-AuPd, the anodic peak current increases up to the 20^th^ cycle; and then, it remains almost unchanged up to the 100^th^ cycle. For AuPd, the anodic peak current increases up to 50^th^ cycle and then it is stable only within 50^th^ and 70^th^ cycle; suggesting the durability of EA-AuPd is considerably higher than that of AuPd through the ADT.^31,56–60^

Chronoamperometric experiment was evaluated by setting the working electrodes at the selected potential in which the electro-oxidation of methanol is warranted. Fig. 8b represents the chronoamperograms of EA-AuPd and AuPd that were recorded in methanol and NaOH solution at the appropriate potential of −0.18 V (*vs.* Ag/AgCl). As expected, the initial decay in current densities is possibly attributed to the accumulation of reaction intermediates during the MOR. Afterwards, the currents gradually decrease and finally achieve to a pseudo steady state condition.^31,59–63^ However, it is noticeably that the EA-AuPd has more than 2.5-fold higher current density over AuPd during the entire period (2000 s) of the test. It is noted that the same issue is obtained from chronoamperometry experiment for AuPd and EA-AuPd in ethanol solution (Fig. S9†). This results demonstrate that at the investigated potential, the EA-AuPd has a better electrochemical stability. All of the above results confirm the electrocatalytic superiority of EA-catalysts towards MOR and EOR.

**Fig. 9:**
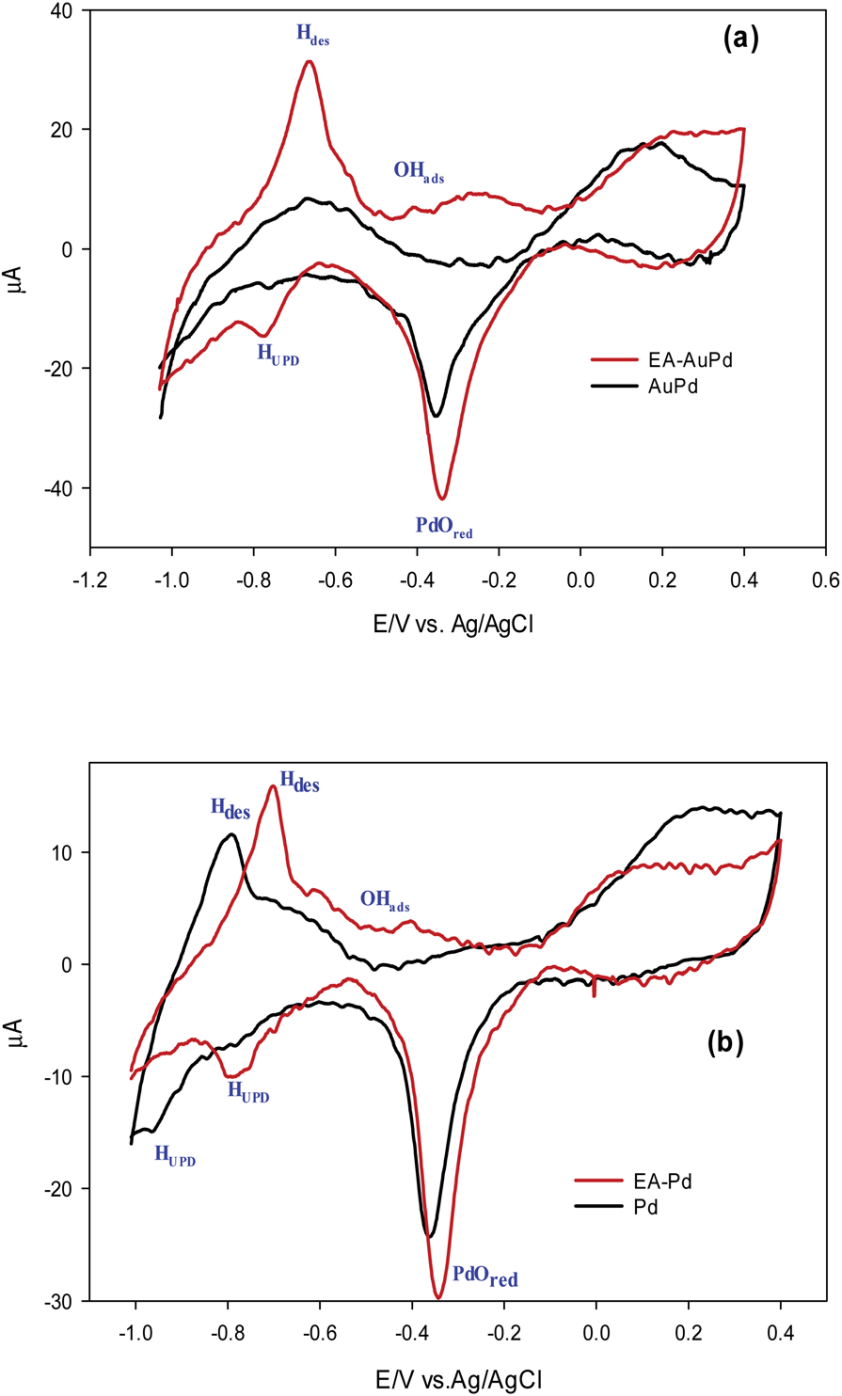
Comparison of CV curves in 1.0 M NaOH solution for the (a) AuPd and (b) Pd, before and after being subjected to EA process (scan rate 50 mV s^−1^).

**Scheme 2:**
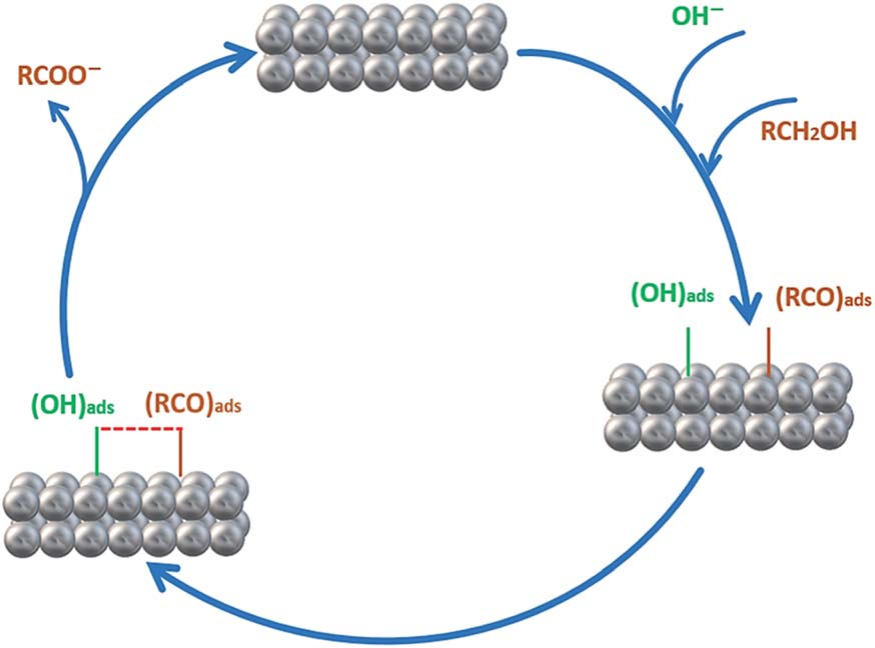
Schematic diagram of the electro-oxidation of primary alcohols (*e.g.*, methanol and ethanol) at the Pd electrode in alkaline media.

In order to investigate the effect of the EA process on MOR and EOR, following discussion is required. Fig. 9a, b and also Fig. S10† show the CVs for AuPd, Pd and Pd/C in 1 M NaOH solution within the potential window of −1 V to 0.4 V, before and after the EA process. Substantial changes occur in the voltammetric response of the electrocatalysts before and after the EA process. The following features must be pointed out: (i) after the EA process, the cathodic peaks in the hydrogen region (potentials below −0.6 V) are appeared in contrast to unclear peaks before the EA treatment, implying that the hydrogen absorption and desorption processes are more favoured at the surfaces of the EA electrocatalysts (ii) the potential in a region between *ca.* −0.6 V to −0.2 V that corresponds to the adsorbed hydroxyl species (OH_ads_) at the surface of the electrocatalyst, show higher currents after the EA process. This could be attributed to the presence of Pd/OH^−^_ent_ that are provided by the EA-process. By the bi-functional theory, the partial and total oxidation of primary alcohols to RCOO^−^ and CO_3_^2−^ needs the adequate OH_ads_ species at the surface of the electrocatalyst in alkaline medium (Scheme 2).^60,64,65^ Based on the well accepted reaction mechanism (eqn (6), (7), (8) and (10)), the Pd-OH_ads_ group is a key reactant in the r.d.s of EOR and MOR (eq. (9)).^48,66–69^ In this regard, due to the presence of Pd/OH^−^_ent_ (from the EA-process), the larger quantities of oxygenated groups are adsorbed at the surface of the electrode and provides more species in terms of Pd–OH_ads_ as evidenced by the improved current density as well as the negative shift of anodic peak potential.

**Fig. 10:**
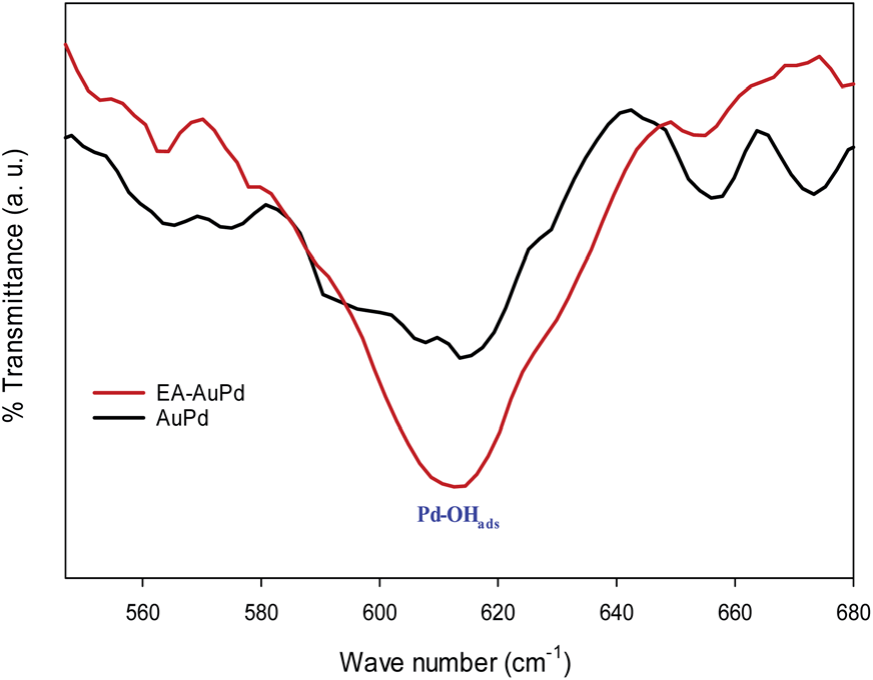
Comparison of FT-IR spectra of AuPd and EA-AuPd after being subjected to cyclic voltammetry in 1.0 M NaOH (potential range: −0.8 V to 0.4 V, scan rate 50 mV s^−1^).

Moreover, in an effort to indicate the increasing of the Pd–OH_ads_ groups after the EA-HER, the FT-IR spectra of the AuPd and EA-AuPd were taken after cyclic voltammetry in 1 M NaOH solution (Fig. 10). The intensity of the band at 620 cm^−1^, which is attributed to the Pd–OH_ads_, is higher for EA-AuPd, verifying the effective role of the EA process in the enhancement of the Pd–OH_ads_ at the surface of the electrocatalyst.^58^

6 Pd + (RCH_2_OH)_sol_ → Pd–(RCH_2_OH)_ads_

7 Pd–(RCH_2_OH)_ads_ + 3OH^−^ → Pd–(RCO)_ads_ + 3H_2_O + 3e^−^

8 Pd + OH^−^ → Pd–OH_ads_ + e^−^

9 Pd−(RCO)_ads_ + Pd−OH_ads_ → Pd−(RCOOH) + Pd

10 RCOOH + OH^−^ → RCOO^−^ +H_2_O

(iii) In the cathodic scan, the current density of PdO reduction peak increases due to the application of EA process with a subtle positive shift in the reduction potential. By calculating the reduction charge of PdO, it was revealed that the electrochemical active surface area^50,70^ (ECSA) has been increased about 20%.

Additionally, to verify the probable morphological surface alternations caused by the EA process, SEM technique was also used. It was found that the surface roughness of AuPd has increased compared to that before the EA process (Fig. S11†). Based on all of the above-mentioned results, it can be concluded that the EA process is in the favour of MOR and EOR.

## Conclusions

In conclusion, an *in situ* cathodic electrochemical activation protocol is provided for the AuPd and Pd electrocatalysts to effectively expedite the HER, MOR and EOR activity in alkaline media. From the represented experimental evidences, we propose that the electrochemical active Pd/OH^−^_ent_ species are generated on the surface of EA-AuPd and EA-Pd electrocatalyst. In the alkaline HER, tuning the surface hydrophilicity was achieved by forming the Pd/OH^−^_ent_⋯OH_2_ activated complex and therefore, promoting the electrocatalytic activity. On the other hand, in alkaline MOR and EOR, Pd/OH^−^_ent_ species facilitate the adsorption of oxygenated groups, especially OH^−^ ions in terms of Pd–OH_ads_, which is a key reactant in MOR and EOR. We believe that this research offers a practical approach to expose the active species for enhanced electrocatalytic applications.

## Conflicts of interest

There are no conflicts to declare.

## Supplementary Material

RA-011-D0RA07817D-s001
