# Supplementary material for: In situ electrochemical activation as a generic strategy for promoting the electrocatalytic hydrogen evolution reaction and alcohol electro-oxidation in alkaline medium
Source: RSC Adv. 2021 Mar 12;11(18):10615–24. doi: 10.1039/d0ra07817d (PMC8695632; doi:10.1039/d0ra07817d)
Supplement: RA-011-D0RA07817D-s001 [file RA-011-D0RA07817D-s001.pdf]

Electronic Supplementary Material (ESI) for RSC Advances.
This journal is © The Royal Society of Chemistry 2021

In-situ electrochemical activation as a generic strategy for promoting electrocatalytic hydrogen evolution reaction and alcohol electro-oxidation in alkaline medium

Alireza Kardan [a], Narges Ashraf [b], Zeynab Dabirifar [a] and Sara Khadempir*[a]

[a] Department of Chemical Engineering, Quchan University of Technology, Quchan, Iran.

[b] Department of Chemistry, Faculty of Sciences, Ferdowsi University of Mashhad, Mashhad, Iran.

.

*Corresponding Author: s.khadempir@qiet.ac.ir (Sara. Khadempir)

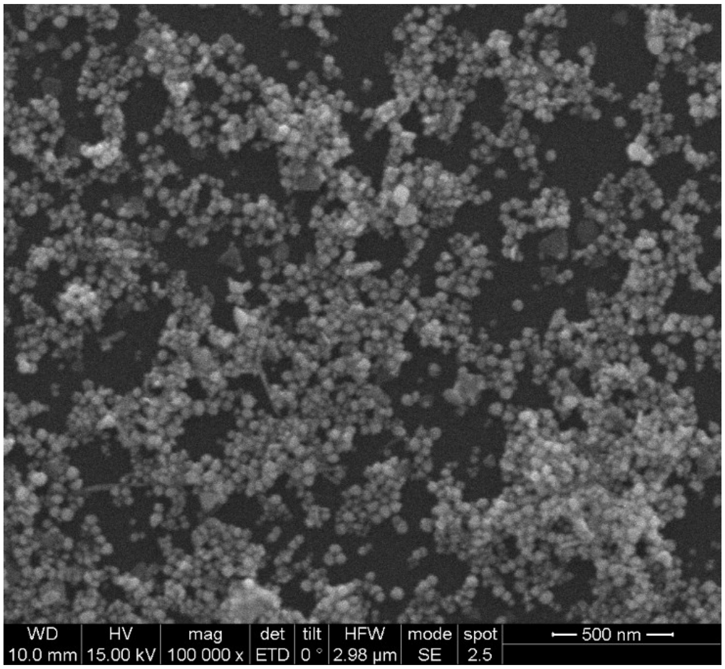

Fig. S1: SEM image of AuPd NPs.

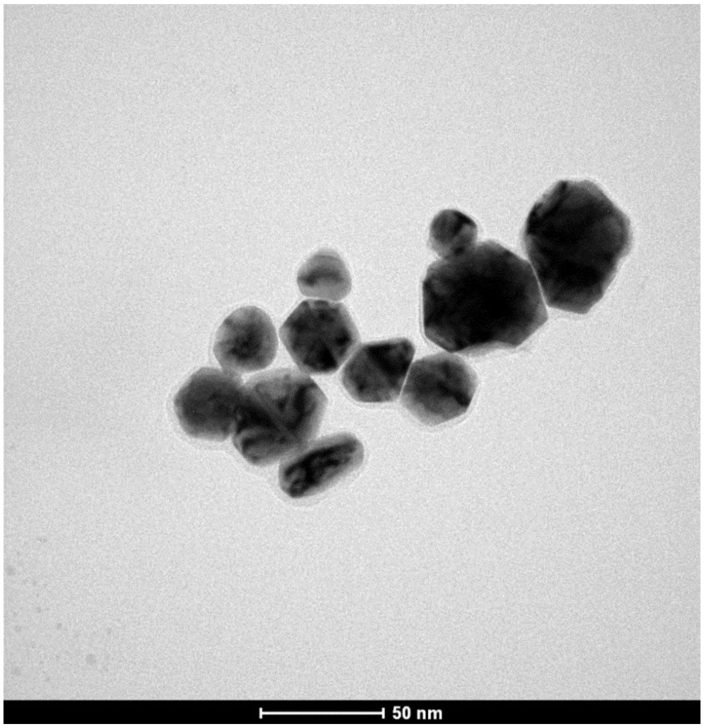

Fig. S2: TEM image of AuPd NPs.

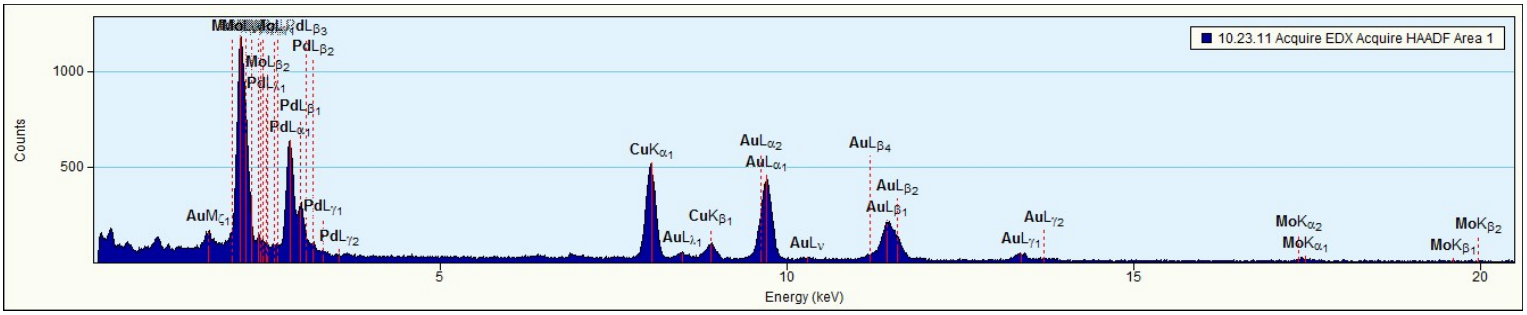

Fjg. S3: EDS spectra of AuPd NPs.

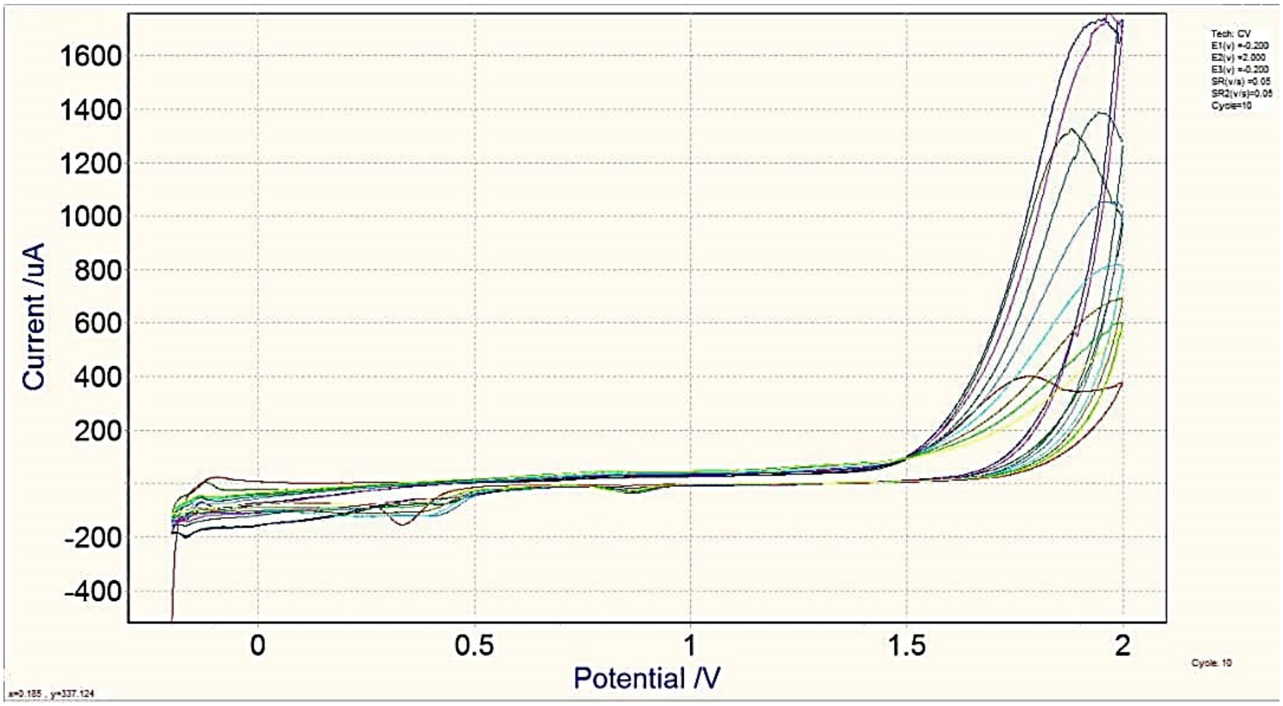

Fig. S4: Cyclic voltammograms (CVs) of AuPd NPs in 0.5 M H_2SO_4 from scan 10 onwards. (Scan rate 50 mV s^{-1})

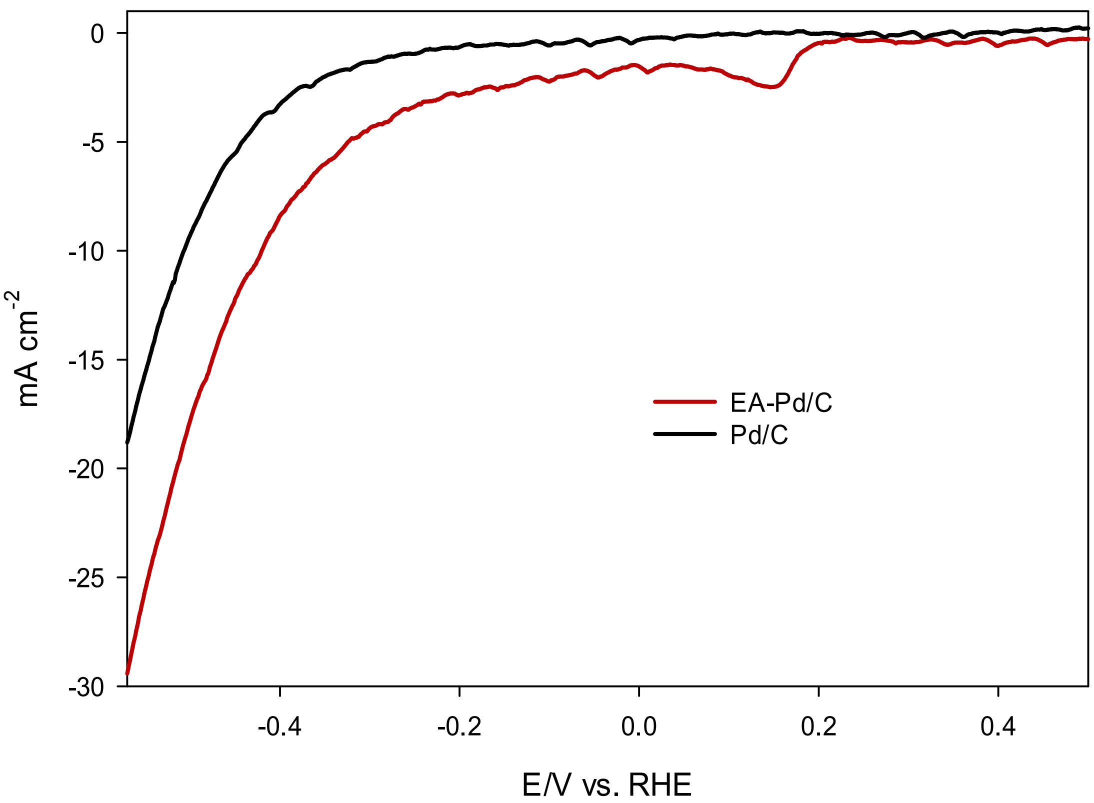

Fig. S5: LSV polarization curves for the HER in 1 M NaOH on Pd/C and EA-Pd/C electrocatalysts.

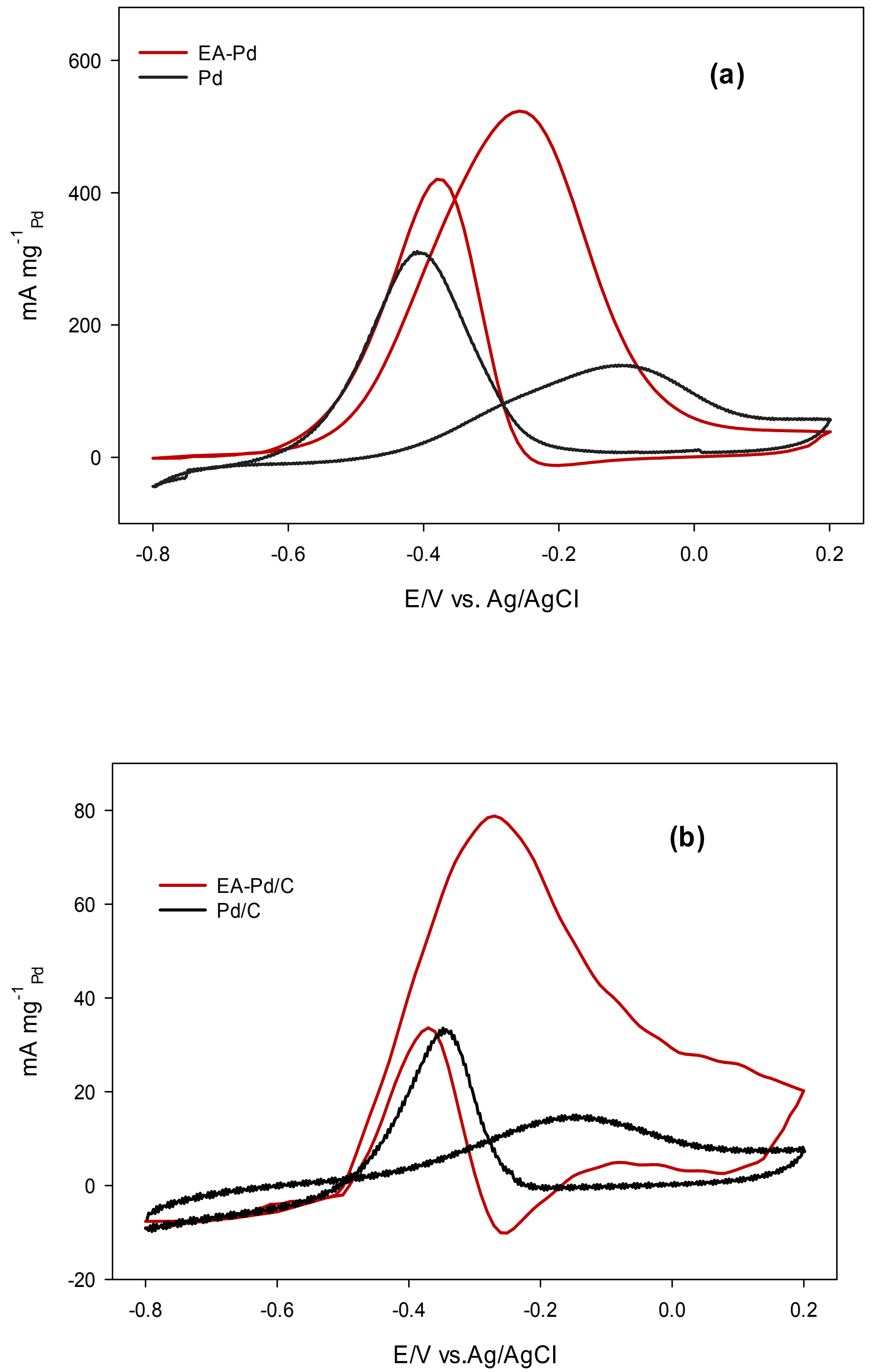

Fig. S6: Voltammetric profile of (a) Pd and EA-Pd, (b) Pd/C and EA-Pd/C. All experiments were conducted in a 1 M aqueous solution of ethanol using 1 M NaOH as the supporting electrolyte at a scan rate of 50 mV s^{-1}.

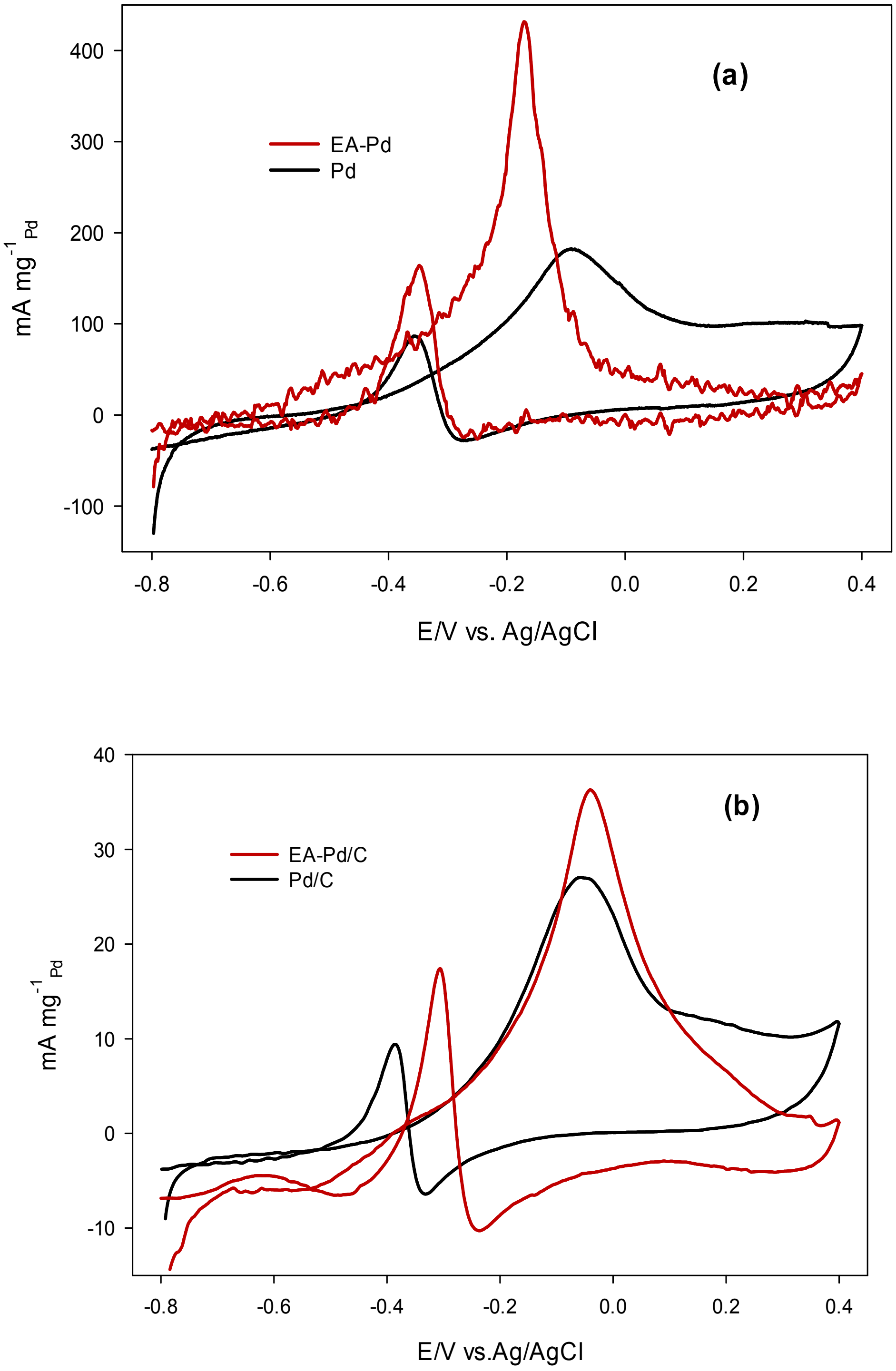

Fig. S7: Voltammetric profile of (a) Pd and EA-Pd, (b) Pd/C and EA-Pd/C. All experiments were conducted in a 1 M aqueous solution of methanol using 1 M NaOH as the supporting electrolyte at a scan rate of 50 mV s^{-1}.

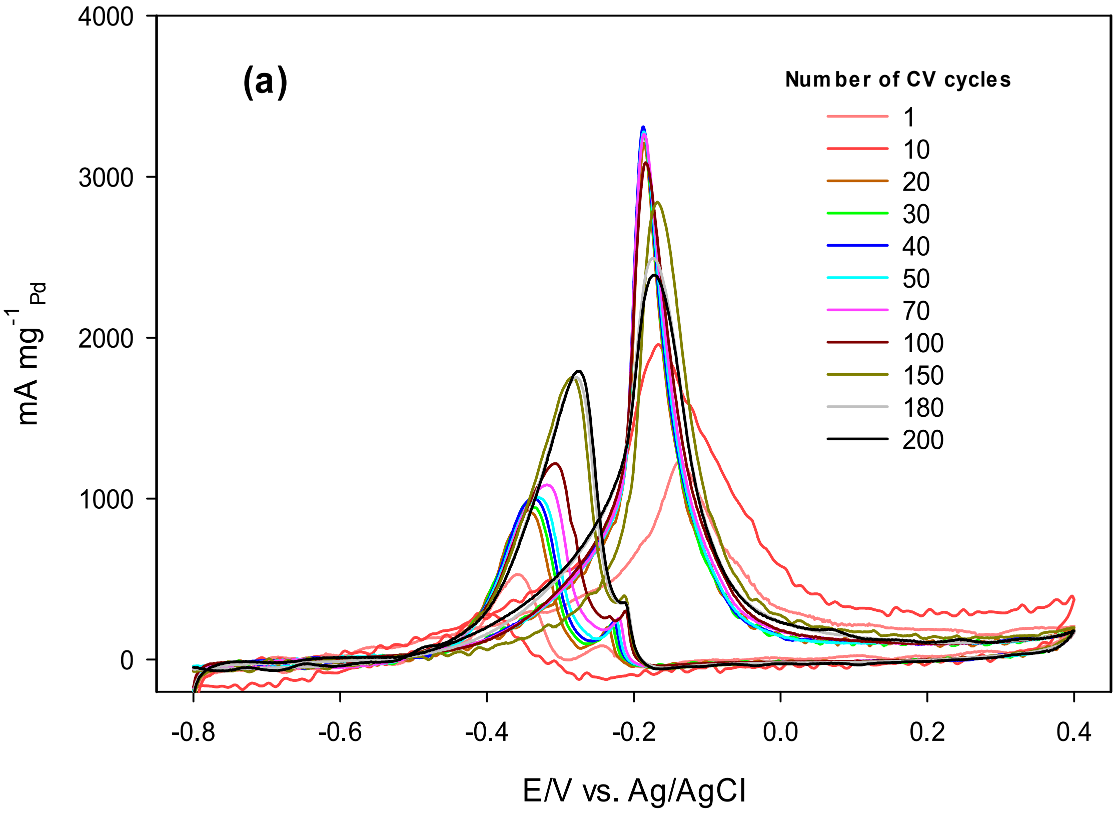

(a)
Number of CV cycles
1
10
20
30
40
50
70
100
150
180
200
4000
3000
2000
1000
0
mA mg^{-1}$_{Pd}$
-0.8
-0.6
-0.4
-0.2
0.0
0.2
0.4
E/V vs. Ag/AgCl

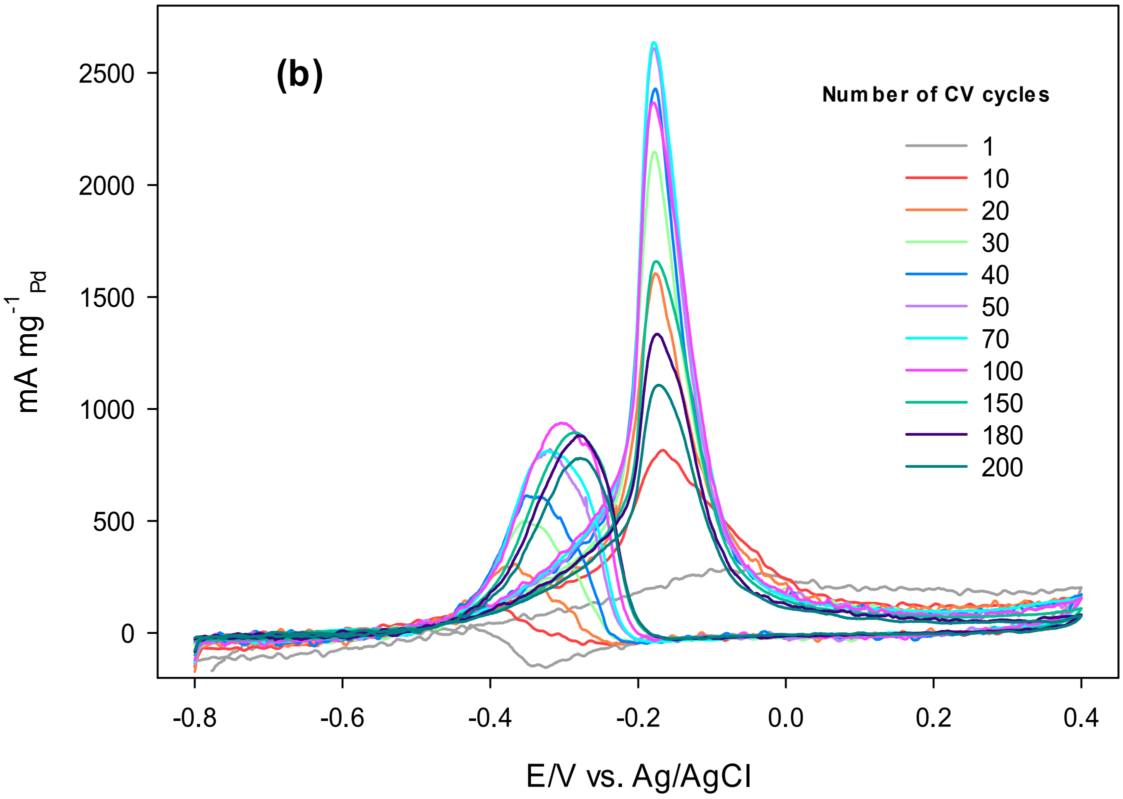

Fig. S8: Two hundred consecutive CV curves measured in 1M methanol and 1 M NaOH at (a) EA-AuPd, (b) AuPd.

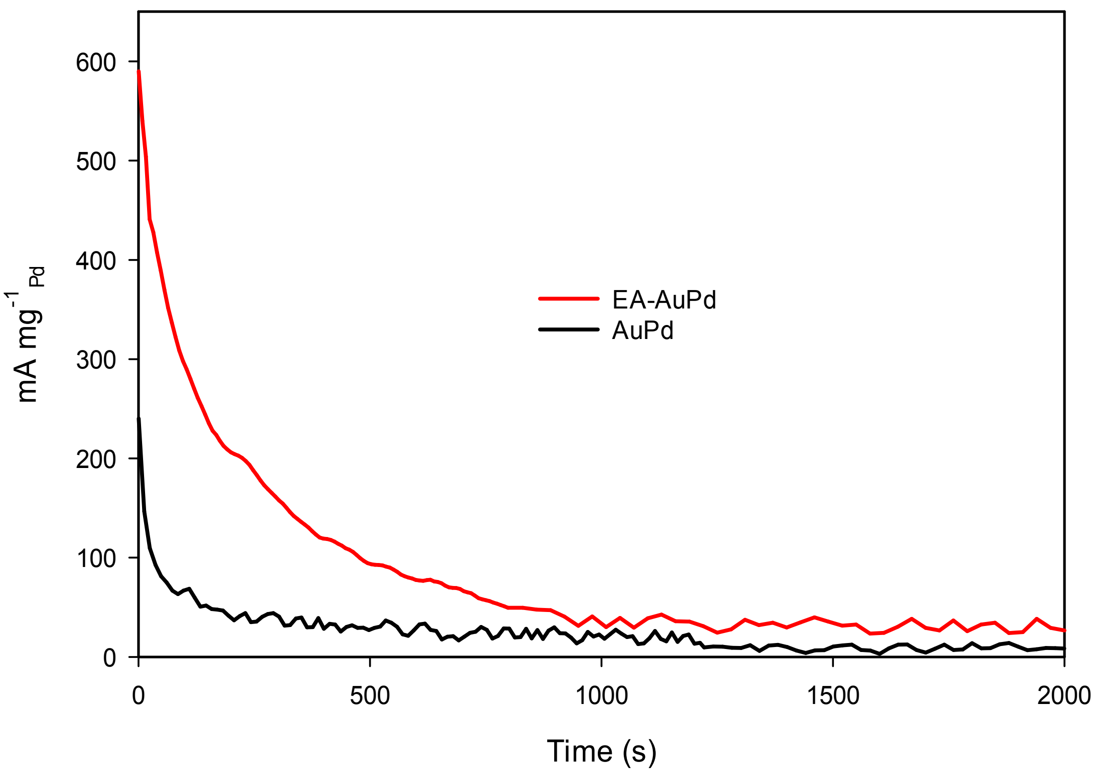

Fig. S9: Chronoamperometry curves of EA-AuPd and AuPd catalysts for the EOR measured at -0.3 V (vs. Ag/AgCl). All experiments were conducted in 1 M ethanol + 1 M NaOH.

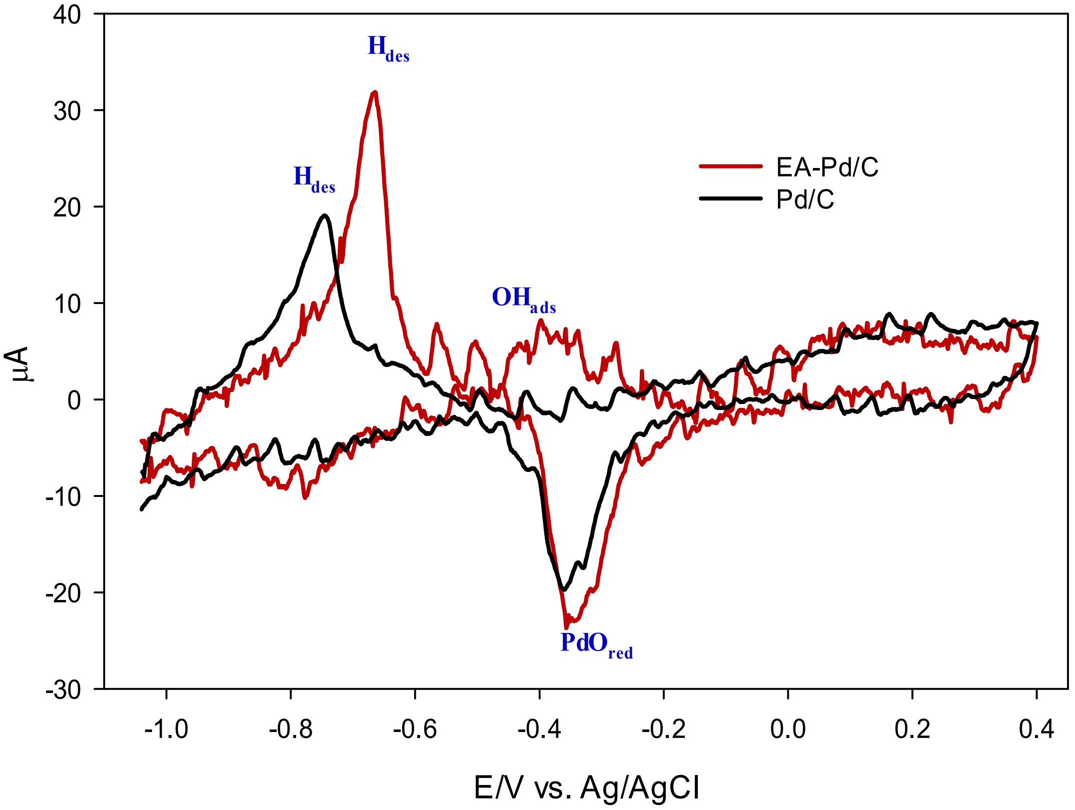

Fig. S10: Comparison of CV curves in 1.0 M NaOH solution for the Pd/C, before and after being subjected to EA process (Scan rate 50 mV s^{-1}).

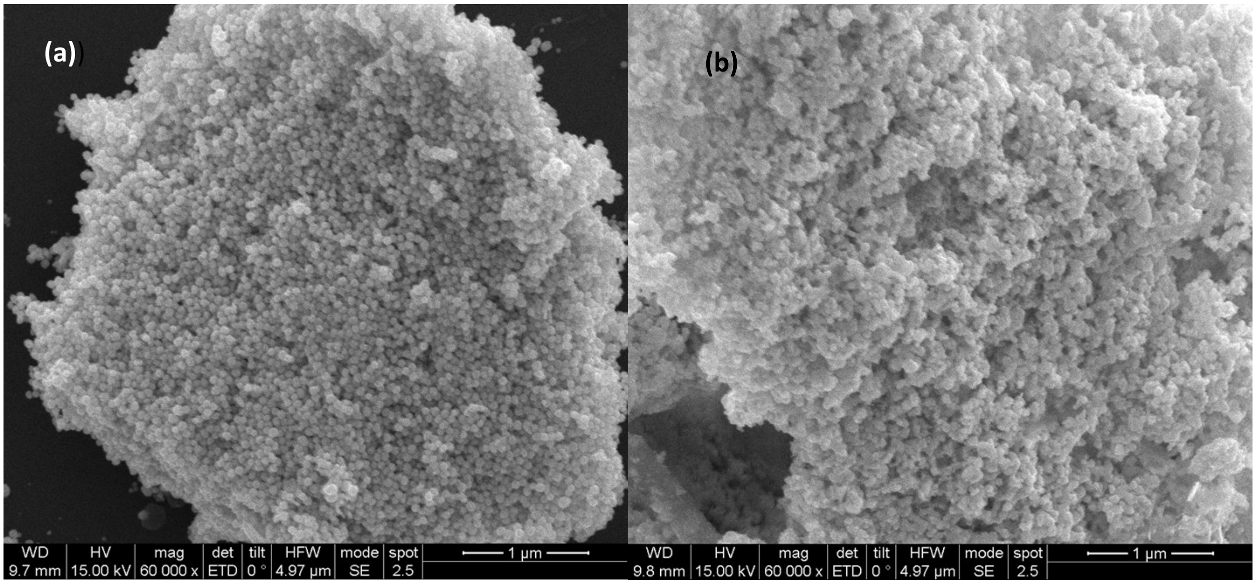

Fig. S11: SEM image of (a) AuPd, (b) EA-AuPd.
